# Gender beliefs and legitimization of dating violence in adolescents

**DOI:** 10.1515/med-2025-1188

**Published:** 2025-04-17

**Authors:** Sónia Brás Ferreira, Mayana Bonfim Ferreira, Nadirlene Pereira Gomes, Amâncio António de Sousa Carvalho

**Affiliations:** Local Health Unit of Tâmega and Sousa, Community Health Care Unit, Baião, Portugal; Federal University of Bahia, Nursing School, Salvador, Bahia State, Brazil; Federal University of Bahia, Nursing School, Salvador, Bahia State, Brazil; Health School, University of Trás-os-Montes and Alto Douro, Quinta de Prados, 5000-801, Vila Real, Portugal; External integrated member of Research Centre on Child Studies, University of Minho, Braga, Portugal

**Keywords:** beliefs, gender studies, violence, adolescent, community health nursing, public health

## Abstract

**Background and aim:**

Knowing the gender beliefs (GB) that legitimize dating violence (DV) it is important for the prevention of this phenomenon. The aim is to evaluate the impact of GB interventions that legitimize DV.

**Methods:**

Single group quasi-experimental study, with a sample of 148 Portuguese adolescents. A questionnaire was used to collect data, with data processing carried out using SPSS, using descriptive and inferential statistics.

**Results:**

The interventions included an infographic on gender asymmetries, a video about DV, and posters on the topic and Health Education sessions. The largest group fell into the less conservative GB (40.5%) and the categories of non-violent relationship and considerably violent relationship had the same percentage (38.5%). The rank mean of the gender belief inventory scale before and after the interventions was, respectively, 35.24 and 33.06 points, while the same measurements of the violent youth relations inventory scale were 2.74 and 1.63 points. There were statistically significant differences (Wilcoxon: *p* = 0.01) between the GB score before and after the interventions, as well as in the violent youth relations (VYR) scale score (Wilcoxon: *p* = 0.000).

**Conclusion:**

The interventions had a significant impact on reducing the GB legitimizing the DV and VYR, and were effective.

## 1 Introduction

The word adolescence derives from the Latin *adolescere*, meaning to grow, to become great [1]. Adolescence is considered the transition period between childhood and adulthood; however, its chronological delimitation differs depending on the sources. The World Health Organization [2] places adolescence at the age of between 10 and 19 years. Other authors [3] consider that a more comprehensive definition of adolescence is necessary, placing this phase of the human life cycle between 10 and 24 years of age. In fact, this vision aligns more with current patterns of growth and maturity, in addition to social standards.

Regarding the duration of adolescence, it is possible to subdivide it into three distinct phases based on chronological age: initial adolescence (between 10 and 13 years old), intermediate adolescence (between 14 and 17 years old), and late adolescence (between 18 and 24 years old). The first phase is characterized by puberty, changes especially at a physical level and reactions to these same changes, while in the second phase there is great social pressure to imitate peers and emphasize appearance and material goods, and in late adolescence, they assume roles closer to adult life [1].

These phases of adolescence do not coincide with the education cycles in Portugal (third cycle). The third cycle of education includes students in the seventh, eighth, and ninth year of schooling, in which students are usually 12, 13, and 14 years old. The study only included ninth grade students (14 years old), and there may be younger students (13 years old) because they were admitted to school early and older than 14 years old, aged between 15 and 17 years old, as they had failed the previous years, from one to three times during their school career.

All these changes require adolescents to make efforts to adapt to new realities, which involve relationships between adolescents and their context, namely with family, school, peers, and the community. This transition, although it marks in a certain way the break with childhood, must be seen in the form of a positive adaptive process, since the adolescent builds their individual beliefs at this stage, dissociates themselves from their family, and progressively acquires psychosocial autonomy [1].

In addition to all these aspects, adolescence is also the period of awakening to sexual attraction and when romantic relationships begin to occur, being a favorable phase for the development of beliefs related to the idealization of love and romantic myths. Thus, the myths of romantic love reside mainly in the obsessive search for the perfect partner, synonymous with complete happiness, and it is common to consider that suffering is natural if such a complement is not found. These authors found evidence that love myths related to the idealization of love are accepted as natural by adolescents. Furthermore, they observed a set of beliefs related to gender roles associated with these romantic myths, in the same way as the normalization of attitudes of violence against the partner was observed in association with the same myths. Their study also revealed that the increase in sexist attitudes, whether hostile or benevolent, appears to lead to the mythification of love. These attitudes and beliefs constitute risk factors for the establishment of unbalanced dating relationships with a tendency toward violence [4].

Achieving gender equality has been one of the biggest challenges faced by societies worldwide, in all spheres of human life. Therefore, the United Nations (UN) included gender equality in the Sustainable Development Goals (SDGs) for the 2030 Agenda, known as SDG5. The 2030 Agenda and the 17 SDGs constitute the common vision for humanity, establishing a contract between world leaders and people to improve the planet and its people [5]. SDG5 includes nine objectives to promote gender equality, as a tool for the sustainable development of the world, the first being essential to guarantee effective equality, as it aims to end all forms of discrimination against all women and girls worldwide.

At Portugal level, it is possible to understand that the goals, in addition to being shared, are extremely important, as, according to the European *ranking Gender Equality Index* [6], Portugal is in 15th place, with 5.8 points below the European *score*. Despite still being in a fragile position compared to other European Union (EU) countries, the Portuguese score has increased since 2010 by 9.1 points, which led to an increase of four places.

Despite the need for many improvements in this area, the majority of adolescents have positive attitudes toward gender equality, while at the same time believing in traditional gender roles [7]. This duality demonstrates that gender inequalities in adolescence derive mainly from the learning of traditional gender roles and characteristics imposed since early childhood, through the repetition of behaviors, concepts, and attitudes that reiterate the authoritarian and dominant leadership attitudes of men and the posture dependent and submissive of women [8]. However, with the evolution of society, the media coverage of inequalities, and inclusion policies, adolescents are receptive to reducing these same inequalities.

In this way, the view that teenagers attribute to gender roles in the context of dating is directly related to the concept of ambivalent sexism, defined by Glick and Fiske in 1996 [9]. This particular type of sexism encompasses two components for women: hostile sexism and benevolent sexism. The first is characterized by the presence of dominating attitudes of boys toward girls, based on the premise of male superiority in economic, political, and social institutions. The second occurs when the man expresses attitudes of protection and affection toward the woman with the aim of highlighting his dependence on the man in the emotional and sexual aspects. This second type of sexism leads to a devaluation of women, which can be as negative as that resulting from hostile sexism.

Ambivalent sexism thus promotes the maintenance of gender stereotypes, which generates deep-rooted inequalities in society. This phenomenon in particular leads to a greater acceptance of violence in dating relationships [9].

Hence, it is possible to understand that many of the beliefs that legitimize violence are related to inequality between men and women, in which men, seen as the element with more power, have the privilege of controlling other members of the family, often with the approval of women, children, and society. These are the models that teenagers have as a reference throughout their growth, leading to the replication of what they have observed and learned over the years [9].

Also Hunt et al. [10] state that attitudes supporting gender inequality in society and relationships can encourage dating violence (DV) victimization. Scientific evidence indicates that maintaining attitudes that promote traditional gender roles perpetuates gender inequality and promotes violence against women.

Thus, in the context of adolescence, support from society in general and peers for traditional gender roles that normalize the more aggressive attitudes of young males in relationships are strong predictors of DV. Studies indicate that this type of legitimation is strongly based on patriarchal culture, being linked to racism, heterosexism, and poverty. Although much of the literature examines perpetration with regard to gender inequality, it is unclear how attitudes toward traditional gender roles and gender inequality are associated with dating victimization in both sexes [10].

This topic is extremely important, as violence in intimate relationships is not, at all, a phenomenon exclusive to relationships between adults. National studies by the Portuguese Victim Support Association and other international studies reveal that this is also a problem in relationships between younger people [11].

In fact, romantic relationships are important throughout the life cycle, contributing to personal well-being, generally starting in adolescence. It is at this stage that adolescents go through the construction of their self and hetero-perceived identity through their peers, as well as a deeper connection with regard to interpersonal relationships outside the household. Since violence can arise in different ways in the context of dating, it is important to know at an earlier stage of adolescence the gender belief (GB) that legitimize DV, as well as the prevalence of victimization in adolescents in the eighth and ninth grades of schooling (third cycle) [12].

According to the Plano i Association [12], in its study called “National study on dating violence in a university context: beliefs and practices – 2017/2020,” around 53.9% of respondents have already suffered at least one act of violence in dating, 35% have participated in at least one act of DV, in addition, 3.6% of women and 15.4% of men agree that jealousy is a proof of love. Other data related to the type of violence carried out were also obtained: 14.5% of women and 11.5% of men have already suffered violence due to blackmail and threats and 16.4% of women and 14.7% of men have had their social networks hacked, mobile phone, email, or other means of communication/interaction. However, little is known about this problem in third-grade adolescents, in particular.

It is within the scope of this problem that this study emerges, with the general objective of evaluating the impact of interventions in GB that legitimize DV on the adolescents in the sample.

## 2 Methods

This is a quasi-experimental, single-group, longitudinal study with a quantitative approach [13]. In terms of study design, we began by making a situation diagnosis (Moment 1 of data collection), using an instrument that includes the Gender belief inventory (GBI) and the violent youth relations inventory (VYRI) in ten classes from three school groups, the context of this study. We then proceed with data processing and analysis. Based on the results found, we planned four interventions that took place between November 25, 2022 and January 17, 2023. After 13 days, we carried out the second data collection (Moment 2), using the same data collection instrument. The data were, again, processed and analyzed and this article is being prepared based on them.

### 2.1 Population and sample

The population was defined using the following inclusion criteria: (i) adolescents enrolled in the 2022/2023 school year, in the ninth year of schooling, in three groups of public schools in the third cycle of a municipality in the North of Portugal; (ii) adolescents aged between 13 and 17 years old. After applying these inclusion criteria and according to data from the General Directorate of Education and Science Statistics (DGEEC, 2021), it was estimated that the population consisted of around 346 students enrolled in the ninth year of public education, from the aforementioned municipality.

Since it was not possible to study the entire population, there was a need to select a representative sample of the population, with the following exclusion criteria being defined: (i) adolescents who did not present informed consent duly signed by their guardian/tutor and (ii) adolescents who did not complete at least 80% of the questionnaire. After applying the defined exclusion criteria, the sample consisted of 148 adolescents, i.e., around 42.8% of the target population. We opted for non-probabilistic sampling, for convenience, which, according to Fortin et al. [14], it consists of elements with easy access, at a specific time and place.

Students enrolled in public education, in the 2022/23 school year, in the ninth year of schooling, in the municipality of the Northern Region of Portugal, involving three school groups, were 346 young people. The ten classes of the three school groups selected for this study were made up of 210 students. To respect ethical principles, we need to obtain the consent of parents/guardians, having asked the students to take them. Therefore, we delivered 210 informed consent forms. During this procedure, only 148 consents were given to us, so the sample consisted of 148 students. None of the students were excluded from the sample for not having completed at least 80% of the questions.

### 2.2 Data collection instrument

For this study, the data collection instrument selected was the questionnaire, as it is a quick-to-complete tool that protects anonymity and is low-cost, which best adapted to the nature of this study. It consisted of three parts: the first part of the questionnaire included sociodemographic data, which allowed the sample to be characterized; the second part included the scale called the GBI, whose objective is to assess the beliefs of the adolescents in the sample about social gender relations; and the third part consisted of the VYRI and consists of two subscales: the first collects data on DV suffered and/or perpetrated, in addition to characterizing the psychological, physical, sexual, and social violence suffered and/or practiced; the second subscale assesses beliefs regarding victimization in the context of DV. Both the GBI like the second subscale of VYRI were subjected to internal consistency assessment for the Portuguese population by Neves et al., within the scope of the National Study on Dating Violence in a University Context: Beliefs and Practices – 2017/2020 [15].

#### 2.2.1 GBI

The GBI has 24 items on a Likert scale, with the following response options: “I don’t agree,” “I neither agree nor disagree,” and “I agree”. The variable “Gender beliefs” (GB) is determined using the sum of the scores of all 24 items, after inverting items GB4, GB9, GB10, GB15, and GB19. The scale score ranges from 24 to 72 points, with higher values corresponding to more conservative beliefs. No boundary value or cutoff points were defined by the scale author. The GBI scale allows us to assess adolescents’ beliefs about gender relations and meets the objectives of this study. This scale was validated for the Portuguese adolescent population, only through the assessment of internal consistency, having obtained a Cronbach’s alpha of 0.742 [15]. The authors of this subscale did not perform factor analysis.

#### 2.2.2 VYRI

The VYRI consists of two subscales. The first consists of 21 items with the following response options: “It’s never happened to me,” “I’ve already suffered,” and “I’ve already done it”. In this subscale: (i) the proportion of violence suffered is determined by counting the number of “I have already suffered” responses in the 21 items and divided by 21 (number of items); (ii) the proportion of violence perpetrated is determined by counting the number of “I have already done” responses in the 21 items and divided by 21 (number of items); (iii) variable “I suffered violence”: 0 – No; 1 – Yes, if you respond to at least 1 item with “I have already suffered,” then “I suffered violence” = 1; (iv) variable “I did violence”: 0 – No; 1 – Yes, if you respond to at least 1 item with “I’ve already done it,” then “I’ve done it violence” = 1. The data are processed according to response proportions, so the values obtained will be in accordance with psychological, physical, sexual, and social victimization and/or perpetration. The items that identify with the different types of violence are (a) psychological violence: items 1, 2, 7, 10, 12; (b) physical violence: items 8, 13, 14, 15, 18; (c) social violence: items 3, 5, 11, 17, 19; (d) sexual violence: items 4, 16, 20, 21; and (e) economic violence: item 6 and (f) persecution: item 9. It is possible to determine the variables indicated from (i) to (iv) for each type of violence, using the items identified from (a) to (e) [15]. The first subscale of the VYRI aims to assess violence suffered and perpetrated, is validated for the population of Portuguese adolescents and is aligned with the objectives of the present study. In this manuscript, only the first subscale will be used. For this first subscale, internal consistency was not assessed, because the response options for the items are not ordinal, but rather dichotomous nominal responses No and Yes.

### 2.3 Data collection

Regarding the application of a questionnaire in a school context, since adolescents are minors, there is a need to obtain duly signed informed consent from their guardians for their participation in the study. After obtaining authorization from the group directors, the present study was approved at the different Pedagogical Council meetings of the three school groups, with the distribution of informed consent statements being the responsibility of the schools, with the collaboration of the class director teachers. They distributed 210 copies of the informed consent to guardians, with 148 duly completed being returned. The discrepancy between the initial number of consents and those returned may be related to an outbreak of influenza A in schools in the municipality, in addition to teacher strikes, not forgetting students’ forgetfulness regarding the delivery of consent. All data for this study were collected through the application of an online, self-administered, and anonymous questionnaire during class hours, ensuring that both teachers and students were informed about its anonymity and confidentiality. Students were free to leave the classroom and withdraw from completing the questionnaire at any time. Data collection was carried out under the supervision of Science or Citizenship teachers, as decided by the respective coordinators of the Health Education Project of each school, accessing it online, through a *Google Forms* ^*®*^ *file*, using students’ computers or their cell phones. Thus, starting from a population of 346 students, we obtained a sample of 148 students, which coincides with the sample size at the first moment of data collection. The first data collection took place from 8 to 25 of November 2022, after verifying and collecting the informed consent statements duly signed by the guardians of 148 students, having answered the questionnaire. The second collection moment took place between January 30 and February 10, 2023, in which 33 students did not complete the questionnaire because they were absent that day, due to illness or because they lost the completion code, which allowed us to pair the cases. The sample was reduced to 115 students, which coincides with the size of the evaluation sample. The researcher was only present at the beginning of this procedure, in order to guarantee the safeguarding of ethical issues and in order to present the study and its objectives, but without interfering with it.

### 2.4 Intervention process

The interventions included the release of a video on DV, entitled “Who loves you, doesn’t attack you,” by the Commission for Citizenship and Gender Equality (Portugal), which was published in the schools’ space, from November 25 to December 16 and from January 9 to 27, 2023, the provision of an infographic on gender inequalities in Portugal, entitled “Asymmetries,” created in the context of this project, released in the week of December 5–9, 2022, the preparation and posting of three posters created exclusively for this project and disseminated within the scope of preventing DV, in the same period of time before, entitled “Dating Violence” aimed at the entire school community and two Health Education sessions in the classes that participated in the project. The first session consisted of a group dynamic on gender equality inspired by the “Earthquake Game” from the CoolBox guide – games for gender equality and non-violence [16], in which content on gender stereotypes and warning signs were covered for violent dating relationships, using the expository, interactive, and role play method, lasting 50 min. The second session consisted of an interactive online game on the Kahoot!^©^ about violence in dating “Do you want me badly or do you love me?,” in which students accessed the online game link, in pairs, using a numerical code, about gender equality and non-violence, using the expository and interactive method, lasting 50 min. The first Health Education session took place on January 10, 2023 and the second session on January 17, 2023. During the study, no external interventions were carried out with the students in the sample, within the scope of the themes of GB and DV.

### 2.5 Data processing

Before starting the analysis itself, the completion of the questionnaires was checked, since one of the exclusion criteria refers to the imperative of completing at least 80% of the questionnaire. In this study, the questionnaires were completed in their entirety, meaning there was no need to exclude any of the cases. Subsequently, a database was built from the questionnaires, where the data were numbered, inserted, grouped, and processed using the *software Statistical Package for the Social Sciences* (SPSS, version 25.0), with statistical analysis carried out using descriptive statistics and inferential statistics. The interpretation of statistical tests was carried out based on a significance level of 5%. Regarding inferential statistics and in the case of nominal measurement variables, frequencies (absolute and relative) and mode were determined. Regarding scalar measurement level variables, in addition to absolute and relative frequencies, measures of central tendency and dispersion were used. In terms of inferential statistics, the normality of the sample was verified using the Kolmogorov–Smirnov and Shapiro–Wilk tests, verifying the homogeneity of variances with the Levene test [17]. To verify the association between the variable sex with the GB and VYR, Student’s *t* parametric test was used, in the case of the association between the age group with GB and VYR the Mann–Whitney test was used, and in the case of the association between the school group with GB and VYR, the ANOVA test was used. To analyze whether there were significant statistical differences between the rank mean of the GB scale score of the adolescents before and after the intervention, we used the Wilcoxon test. The Wilcoxon test, among the tests for non-parametric paired samples, within the scope of bivariate analysis is recommended. Using a multivariate analysis would have been better to eliminate the effects of extraneous variables and potential biases. This was not possible because the study design did not predict these variables [18].


**Ethical approval:** In developing this study, the ethical principles inherent to the Declaration of Helsinki and the Oviedo Convention were respected. To this end, requests for authorization to carry out the study were made to the different directors of the grouped and non-grouped schools chosen to take part in the study, having obtained a favorable opinion from all of them. After obtaining the aforementioned authorizations from the School Management, a request for an opinion was made to the UTAD Ethics Committee, which received a favorable opinion (Ref. Doc84-CE-UTAD-2022 of 19-10-2022).

## 3 Results

Of the total sample (*n* = 148 students, Moment 1), the majority of adolescents were female (52.0%), fell into the 13–14 age group (87.8%), lived in urban areas (53.4%), and the largest group belonged to school group B (36.5%) (Table 1). The average age of the students was 14.05 ± 0.604 years, the minimum value was 13 years and the maximum was 17 years old (data not included in the table).

**Table 1:** Sociodemographic characterization of the sample

Variables	*n*	Percentage
**Sex**		
Masculine	69	46.6
Feminine	77	52.0
Rather not answer	2	1.4
**Age**		
Between 13 and 14 years old	130	87.8
Between 15 and 17 years old	18	12.2
**Housing environment**		
Urban environment	79	53.4
Countryside	69	46.6
**School grouping**		
Group A	42	28.4
Group B	54	36.5
Group C	52	35.1

Of the responses obtained regarding the GBI scale, those obtained in four items stand out, which highlight the need for clarification and deconstruction regarding the vision of the roles attributed to the gender of the adolescents in the sample. The first item with a higher response rate than the rest was item 15 “Women are as violent as men,” in which the majority of adolescents questioned reported neither agreeing nor disagreeing (60.1%), followed by item 1 “The family should be a priority for women,” in which the majority of the sample neither agreed nor disagreed (47.3%) and, in third place, item 17 “Jealousy is a proof of love,” with 40.5% of those in the sample indicated that they neither agree nor disagree and 33.1% agreed with the statement. The responses obtained in relation to this last statement highlight the need to deconstruct the myth of romantic love associated with jealousy, taking into account that 73.6% of the adolescents in the sample chose the wrong answer option.

Regarding the results that show less conservative GB, the answers relating to item 4 “Men can be as good fathers as women can be good mothers” stand out, in which 96.6% of adolescents agreed with the statement, to item 19 “Men and women must have equal rights and duties” in which 92.6% of the sample chose to agree and, finally, to item 9 “Men and women must share household tasks” in which the option “Agree” was chosen by 91.9% of adolescents, which reveals that in the fundamental issues of rights and roles attributed to gender, the majority of adolescents who participated in the study understood gender equality as essential and normalized (Table 2).

**Table 2:** Percentage of each response option obtained for each item on the GBI scale

GBI scale items	I do not agree (%)	I do not agree nor disagree (%)	I agree (%)
1. The family must be a priority for women	39.9	47.3	12.8
2. Women who behave badly should be punished by their partners	89.9	7.4	2.7
3. Women who do not want to be mothers are not true women	89.9	9.5	0.7
4. Men can be just as good fathers as women can be good mothers	0.7	2.7	96.6
5. Women appreciate aggressive men	73.6	23.6	2.7
6. If women did not work outside the home, children would be better educated	70.9	25.7	3.4
7. Women are victims of sexual violence because they provoke men	83.8	11.5	4.7
8. A woman who invests more in her career than in her family is neither a good mother nor a good companion	53.4	35.8	10.8
9. Men and women must share household tasks	4.1	4.1	91.9
10. Men must take parental leave	29.1	27.7	43.2
11. It is gratifying for women to hear jokes	77.0	17.6	5.4
12. Men have more skills than women for leadership positions	64.2	26.3	9.5
13. Women should be more modest than men	75.0	20.9	4.1
14. Some situations of domestic violence are caused by women	45.3	29.0	25.7
15. Women are as violent as men	16.9	60.1	23.0
16. Boys and girls must be educated differently	81.1	11.5	7.4
17. Jealousy is a proof of love	26.4	40.5	33.1
18. Women are more sensitive than men	24.3	39.9	35.8
19. Men and women must have equal rights and duties	3.4	4.1	92.6
20. Men must assume leadership of the family	58.1	34.5	7.4
21. Women who have positions of power behave like men	60.1	33.1	6.8
22. Domestic violence is a problem that must be resolved at home	66.2	20.3	13.5
23. Every woman’s dream is to get married	52.7	41.2	6.1
24. Women who stay in violent love relationships are masochists	65.5	24.3	10.1

The mean score on the GBI scale, at the first moment of data collection, differed significantly between students of different genders (Student’s *t*: *p* = 0.000), with boys obtaining a higher mean than girls (43.32 > 38.75 points). In other words, the boys held more conservative beliefs.

No statistically significant differences were observed between the mean ranking of the GBI scale score among students with different age groups (Mann–Whitney: *p* = 0.381), as well as between the mean GBI scale score of students from different school groups (ANOVA: *p* = 0.056).

Violence in dating relationships was assessed through the application of the first subscale of the VYRI. The answer option “Never happened to me” was mostly marked in all items. However, checking the answers in order to understand the existing victimization, it is possible to verify that the items in which the answer option “I have already suffered” was chosen most times were item 1 “Blame, criticize, insult, defame, accuse without reason” (38.6%), item 12 “Ignore, despise, humiliate, embarrass or treat with indifference” (22.3%), and item 7 “Messing with personal things without authorization (e.g., email account, profile of social networks, coat pockets, cell phone, wallet, diary)” (17.5%), classified as psychological violence and item 17 “Saying bad things or starting rumors at school or in places or groups regularly frequented” (18.9%), as a form of social violence.

In turn, the least marked items in the response option “I’ve already done it” and “I’ve already suffered and I’ve already done it” were item 6 “Keeping all the money or limiting/controlling personal expenses” with 0.6% of responses, item 13 “Threatening or injuring using weapons (e.g., knife, baton, pistol) and/or physical force” and item 18 “Strangling, asphyxiating, running over or seriously injuring or attempting to do so” with 0.7% of responses, obtained, respectively, indicate that, although there are cases of aggravated economic and physical violence, these rarely occurred in the sample under study, so any type of intervention in the community will essentially be from the perspective of primary prevention.

Regarding violence perpetrated (response option “I’ve done it”), the items with the highest number of responses were item 14 “Physically hurting, pushing, kicking, slapping or punching or headbutting” (6.0% of responses “I’ve done *it”* marked), followed by item 1 “Blame, criticize, insult, defame, accuse without reason” with 4.7% of responses, item 3 “Do not allow them to work, study and/or go out alone” (3.4% of the participants responded that they have already done it), and finally with 2.7% of the answers “I *have already done it”* marked, respectively, there is item 7 “Messing with personal things without authorization (e.g., email account, profile social networks, coat pockets, cell phone, wallet, diary)” and item 10 “Threatening verbally or through behaviors that cause fear (e.g., shouting, breaking objects, tearing clothes)”. Thus, with regard to perpetration, the most frequently mentioned form of violence is physical violence, followed by psychological violence and social violence.

Item 5 “Prevent contact with family, friends, neighbors (e.g., prohibit speaking to anyone, take away or turn off your cell phone)” and item 20 “Forcing sexual intercourse” with 0.0% of the answers, respectively, i.e., they were not marked by the students in the sample, which indicates that in the context of our sample, sexual violence and isolation (social violence) did not constitute forms of violence exercised (Table 3).

**Table 3:** Percentage of response options obtained in the first subscale of the VYRI

VYRI scale items	Never happened to me (%)	I have already suffered (%)	I have done (%)	I have already suffered and I have already done (%)
1. Blame, criticize, insult, defame, accuse without reason	54.7	38.6	4.7	2.0
2. Using communication technologies to threaten or blackmail (e.g., WhatsApp, Instagram, Facebook, cell phone, email or others)	88.5	10.8	0.7	0.0
3. Do not allow them to work, study and/or go out alone	93.2	2.7	3.4	0.7
4. Forcing unwanted sexual behavior (e.g., watching pornography, having oral sex, having anal sex or having sexual relations with other people)	96.6	2.7	0.7	0.0
5. Prevent contact with family, friends, neighbors (e.g., prohibit speaking to anyone, taking away or turning off your cell phone)	94.6	5.4	0.0	0.0
6. Keep all the money or limit/control personal expenses	98.0	0.6	1.4	0.0
7. Touching personal things without authorization (e.g., email account, social media profile, coat pockets, cell phone, wallet, diary)	79.1	17.5	2.7	0.7
8. Make death threats, attempt against life or cause injuries that require medical treatment	93.9	5.4	0.7	0.0
9. Appearing suddenly in public places to monitor or control (e.g., school) and/or pursue (e.g., on foot, in a car or on a motorbike) or ask others to do so	95.9	2.7	1.4	0.0
10. Threatening verbally or through behaviors that cause fear (e.g., shouting, breaking objects, tearing clothes)	83.1	12.8	2.7	1.4
11. Control the way you dress, your hairstyle or image, the places you frequent or your friendships or company	88.5	10.1	1.4	0.0
12. Ignore, despise, humiliate, embarrass, or treat with indifference	74.3	22.3	2.0	1.4
13. Threatening or injuring using weapons (e.g., knife, baton, pistol) and/or physical force	98.6	0.7	0.7	0.0
14. Physically hurting, pushing, kicking, slapping or punching or headbutting	81.8	10.8	6.0	1.4
15. Physically hurt or threaten to hurt people close to you (e.g.	93.2	3.4	2.7	0.7
16. Distribute personal images or videos of a sexual nature without consent	98.0	1.4	0.7	0.0
17. Saying bad things or starting rumors at school or in places or groups regularly frequented	79.7	18.9	0.7	0.7
18. Strangling, suffocating, running over or seriously injuring or attempting to do so	98.6	0.7	0.7	0.0
19. Threatening, defaming or attacking anyone who reports or expresses a desire to report the violence suffered to third parties	97.3	1.3	1.4	0.0
20. Forcing to have sexual intercourse	96.6	3.4	0.0	0.0
21. Threatening to publicly disclose sexual orientation	93.9	5.4	0.7	0.0

We recoded the response options to the first subscale of the VYRI by adding the response options to the items, in which the No option was assigned a score of zero and the Yes option one point, in which the highest score corresponds to greater violence suffered and/or perpetrated.

No statistically significant differences were found between the mean score of the VYRI subscale of students of different genders (Student’s *t*: *p* = 0.679), nor between the mean ranking of the VYRI subscale of students of different age groups (Mann–Whitney: *p* = 0.255), nor between the mean score of students from different school groups (ANOVA: *p* = 0.204).

The majority of students in our sample reported not having a dating relationship (51.4%), denying dating, or ever having a boyfriend. Regarding violence suffered, the majority of the sample reported having already been a victim (56.1%) and not having perpetrated DV (79.1%).

When analyzing DV victimization, psychological violence was the type of violence most reported by the adolescents in the sample (50.0%), followed by social violence (28.5%). Students could indicate several types of violence simultaneously, so the percentages obtained are not cumulative. With regard to perpetrated violence, psychological violence practiced by 64.5% of adolescent perpetrators also stands out, followed by physical violence (45.2%) and social violence (38.7%).

The rank mean of the GBI scale before interventions was 35.24 points and after the interventions was 33.06 points. There were significant statistical differences between the Rank Means of GBI of the adolescents in the sample before (Moment 1) and after the interventions (Moment 2) (Wilcoxon: *p* = 0.01), with 25% of the adolescents reducing their score on the GBI scale. These adolescents were those who had the highest scores, i.e., those students with more conservative beliefs (Table 4).

**Table 4:** Relationship between the sample’s GB score before and after the interventions

Dimension M2 – Dimension M1		*N*	Mean/rank mean	Test value	*p* value	Proportion (%)
M2 GB – M1 GB	Negative ratings	29	23.55			25.00
	Positive ratings	15	20.47	Wilcoxon = −2.372	0.01	13.00
	Ties	71				62.00

Negative ratings – M2 g GB < M1 GB; positive ratings – M2 GB > M1 GB; ties – M2 GB = M1 GB; *p* – probability.

In the GBI subscale, which assesses the level of conservatism of GB, the subscale score decreased from the moment before the intervention (Moment 1) to the moment after the intervention (Moment 2). This decrease is mainly due to the decrease in percentage values in item 15 “Women are as violent as men,” item 1 “The family should be a priority for women,” and item 17 “Jealousy is a proof of love,” in which the largest group in the sample responded “Neither agreed nor disagreed” whose percentages decreased, respectively, to 60.0, 40.9, and 17.4% and in the response option “I agree,” in which the same items obtained a percentage value, respectively, of 18.3, 7.8, and 7.8%, which indicate a decrease in conservatism regarding these items.

Regarding the VYRI subscale, which assesses the violence suffered and perpetrated, the mean rank of the VYRI scale before interventions was 2.74 and after interventions was 1.63 points, decreasing from Moment 1 to Moment 2. This decrease is visible in the percentage values of item 1 “Blame, criticize, insult, defame, accuse without reason,” item 12 “Ignore, belittle, humiliate, embarrass or treat with indifference,” and item 7 “Messing with personal things without authorization (e.g., email account, profile of social networks, coat pockets, cell phone, wallet, diary),” whose percentage values in the answer option “I have already suffered” decreased, respectively, to 20.0, 14.8, and 8.7% and in the answer option “I have done,” whose percentage values also decreased, being, respectively, 4.3, 0.9, and 0.0%. This decrease in GBI and VYRI subscale scores and proportions may be due to the interventions carried out.

There were significant statistical differences between the Rank Means of VYRI score of the adolescents in the sample before (Moment 1) and after the interventions (Moment 2) (Wilcoxon: *p* = 0.000), with 46% of the adolescents reducing their score on the VYRI score scale. These adolescents were those who had the highest score also, i.e., those students with more violence suffered and/or perpetrated (Table 5).

**Table 5:** Relationship between the sample’s VYRI score before and after the interventions

Dimension M2 – Dimension M1		*N*	Mean/rank mean	Test value	*p* value	Proportion (%)
M2 VYRI score – M1 VYRI score	Negative ratings	53	37.75			46.00
	Positive ratings	18	30.86	Wilcoxon = −4.174	0.00	16.00
	Ties	44				38.00

Negative ratings – M2 GB < M1 GB; positive ratings – M2 GB > M1 GB; ties – M2 GB = M1 GB; *p* – probability.

## 4 Discussion

With regard to GB, these results are considered to be very worrying, as it would be expected that social changes and the reduction of the gap between gender roles in society would be translated in a more impactful way in such a young sample. Furthermore, scientific evidence demonstrates that more conservative or sexist attitudes and beliefs increase the risk of legitimizing DV, especially in relation to myths of romantic love and benevolent sexism [19].

In turn, Linares et al. [20] report that there is an association between more conservative GB and psychological violence on the internet exercised against their partners by male teenagers, especially in terms of romantic jealousy and other traditional forms of psychological violence. Thus, young people, influenced by certain myths about romantic love, would start to consider jealousy and control over their partner as an expression of love, which makes it difficult to recognize that certain behaviors are forms of violence and promotes the legitimization of DV. In the present study, when evaluating the degree of agreement with the statement “jealousy is a proof of love,” 33.1% of the adolescents in the sample responded that they agreed with the statement, which may translate into an attitude of legitimization from DV. Taking into account the issue of the romanticization of jealousy, this view of romantic love can lead to legitimizing DV on a psychological level related to these issues.

A study carried out in mainland Portugal and Autonomous Regions [21], with a sample of 5,916 young people between 11 and 25 years old identified that young men legitimized all forms of violence more, in line with the present study.

For Cuadrado-Gordillo et al. [9], the idealization that some adolescents have of romantic relationships justifies violent behaviors based on the myths of “romantic love,” and behaviors such as jealousy, psychological control, or emotional blackmail, is mainly perpetuated by female adolescents, these being behaviors almost as desired. This type of beliefs seems to justify violence and normalize it in social terms, which results in a normalization of violence as a normal way of interacting within the couple, making abuse invisible.

In the present study, the majority of adolescents reported not having a dating relationship (51.4%), which does not match the results obtained by a study carried out in the Northeast of Brazil (Pernambuco – Brazil) [22], with a sample of 270 young people between 12 and 19 years old, in which the vast majority of the sample (83.3%) reported having dating relationships. This difference could be explained by cultural differences between the samples being compared and by the fact that young Brazilians are older.

Regarding the violence suffered in this study, the percentage of victims was 56.1%, while in the study carried out at national level [21], this percentage of young people who were victims of some form of violence perpetrated by their peers was 65.2%, being more expressive than in the present study. Comparing our results regarding victimization with those obtained in the study carried out in the Brazilian Northeast [22], we found that the predominant type of violence suffered is psychological violence (50.0%), which converges with the study in the Brazilian Northeast [22], although the percentage obtained in this study is much higher (81.8%).

With regard to perpetrated violence, the most perpetrated type of violence in this study was psychological violence (64.5%), followed by physical violence (45.2%), much higher percentages than in the national study [21], in which these percentages were 45.1 and 12.2%, respectively, a difference that could be due to the different age, with the study sample being older in comparison. However, the predominant type of violence coincides in both studies. When comparing the same results relating to this study with those obtained by the Brazilian Northeast Study [22], it is clear that the findings of this study record a higher percentage in psychological violence (86.2%), but lower in the type of physical violence (37.6%), with the predominance of psychological violence remaining.

The results obtained regarding DV victimization in the sample are consistent with the findings of Farmer et al. [23] in their meta-analysis, involving DV in a school context, since these authors found that the prevalence of DV was between a quarter and a third of adolescents in studies from several Western countries, leaving, however, a possibility for these values reach around two thirds of students, depending on the context. In relation to the present study, VYR constitute more than half of the sample (61.5% of the responses obtained), i.e., practically double the minimum prevalence in the study by Farmer et al. [23] and very close to the maximum estimate of two-thirds.

In the study by Mineo et al. [24], there are differences regarding psychological violence, which affected 16% of the sample, in contrast to 50% of the adolescents in the sample of the present study, who reported having suffered this form of DV. However, the issue of sexual violence in the present study represents less than half of the results obtained by those authors in a study on DV in Italian adolescents. This can be explained by the fact that the study in comparison included sexual offenses at a verbal level, which in the questionnaire used in this study is not explicitly covered, i.e., in the applied scale (VYRI) it was only asked about the dissemination of images of a sexual nature on the internet and disclosing one’s sexual orientation to other people.

The GBI scale score decreased after the interventions in 25% of the adolescents in the sample as opposed to 13% of adolescents in which it increased, which means that in the largest group of adolescents in the sample the GB became less conservative and legitimizing DV. The VYRI subscale score also decreased after the interventions in 46% of the adolescents of the sample as opposed to 16% of adolescents in which it increased, meaning that the violence suffered and/or perpetrated appears to have decreased. These results allow us to conclude, with some caution due to study limitations that the interventions carried out may have had a positive impact on reducing the conservative beliefs of the adolescents in this sample and the violence suffered and/or perpetrated, while safeguarding the uncertainty of these conclusions due to the limitations of the study.

The main limitations of this study may be the fact that there was no control group, and there may have been interference from extraneous variables in the impact of the interventions performed and the use of a control group will allow obtaining a higher level of scientific evidence. Another limitation is related to the fact that it was a convenience sample (non-random), which may have affected the generalization of the sample results to our population.

This study may have implications for the professional practice of the nursing team, who do school health, which will now have a diagnosis of the situation with regard to GB and VYR and develop more targeted and effective interventions in the context of VYR prevention. These interventions may also allow these adolescents to experience healthier sexuality.

## References

[1] Ramos AL, Barbieri-Figueiredo MC. Enfermagem em Saúde da Criança e do Jovem. 1st edn. Lisboa: Lidel (PT); 2020.

[2] World Health Organization. WHO. Global status report on violence prevention 2014. 2014 [Cited 2025 February 12]. https://www.who.int/publications/i/item/9789241564793#:∼:text=The%20Global%20status%20report%20on%20violence%20prevention%202014%2C,intimate%20partner%20and%20sexual%20violence%2C%20and%20elder%20abuse.

[3] Sawyer SM, Azzopardi PS, Wickremarathne D, Patton GC. The age of adolescence. Lancet Child Adolesc Health. 2018;2(3):223–8. 10.1016/s2352-4642(18)30022-1. 30169257

[4] Martín-Salvador A, Saddiki-Mimoun K, Pérez-Morente MÁ, Álvarez-Serrano MA, Gázquez-López M, Martínez-García E, et al. Dating violence: idealization of love and romantic myths in spanish adolescents. Int J Environ Res Public Health. 2021;18(10):5296. 10.3390/ijerph18105296. PMC8156746 34065736

[5] United Nations Organization. UNO. Transforming our world: The 2030 agenda for sustainable development. 2015 [Cited 2024 2024 March 09]. https://sustainabledevelopment.un.org/content/documents/21252030%20Agenda%20for%20Sustainable%20Development%20web.pdf.

[6] European Institute for Gender Equality. Gender equality index. 2023 [Cited 2024 2024 March 09]. https://eige.europa.eu/gender-equality-index/2022/country/PT.

[7] Kamke K, Widman L, Javidi H. The multidimensionality of adolescent girls’ gender attitudes. Gend Issues. 2021;39(2):236–51. 10.1007/s12147-021-09288-1.

[8] Blázquez-Alonso M, Moreno-Manso JM, Fernández de la Cruz M, García-Baamonde MaE, Guerrero-Molina M, Godoy-Merino MaJ. Psychological inertia in adolescence: sexist attitudes and cognitive and social strategies that hinder gender equality. Curr Psychol. 2019;40(8):3671–81. 10.1007/s12144-019-00327-5.

[9] Cuadrado-Gordillo I, Martín-Mora-Parra G. Influence of cross-cultural factors about sexism, perception of severity, victimization, and gender violence in adolescent dating relationships. Int J Environ Res Public Health. 2022;19(16):10356. 10.3390/ijerph191610356. PMC9408226 36011995

[10] Hunt KE, Robinson LE, Valido A, Espelage DL, Hong JS. Teen dating violence victimization: associations among peer justification, attitudes toward gender inequality, sexual activity, and peer victimization. J Interpers Violence. 2022;37(9–10):5914–36. 10.1177/08862605221085015. PMC9092916 35410536

[11] Associação Portuguesa de Apoio à Vítima. APAV. Folha Informativa: Violência no Namoro. 2020 [Cited 2024 2024 March 09]. https://apav.pt/apav_v3/index.php/pt/.

[12] Neves S, Ferreira M, Borges J, Correia M, Abreu AL, Correia A, et al. Estudo nacional sobre a violência no namoro em contexto universitário: Crenças e práticas – 2017/2020. Associação Plano i; 2020. [Cited 2024 2024 March 04] https://drive.google.com/file/d/1wLwLBSLtJelQOs4QxIXymKUAueUurZBi/view.

[13] Vilelas J. Investigação - o processo de construção do conhecimento. 3st edn. Lisboa: Edições Sílabo (PT); 2020.

[14] Fortin M-F, Côté J, Filion F. Fundamentos e etapas do processo de investigação. 1st edn. Lisboa: Lusodidacta (PT); 2009.

[15] Neves S, Ferreira M, Borges J, Correia M, Abreu AL, Correia A, et al. Inventário das Crenças de Género e Inventário das relações Juvenis Violentas (Versão para Investigação). Maia: Universidade da Maia e Centro Interdisciplinar de Estudos de Género; 2020 [Cited 2024 2024 March 09].

[16] Carreira R, Rojão G. Coolbox - Jogos para a igualdade de género e a não-violência. Lisboa: Coolabora (PT). CRL; 2018.

[17] Melo P. Enfermagem de saúde comunitária e de saúde pública. 1st edn. Lisboa: Lidel (PT); 2020.

[18] Marôco J. Análise estatística com o SPSS statistics. 8th edn. Pêro Pinheiro: Report Number (PT); 2021.

[19] Fernández-Antelo I, Cuadrado-Gordillo I, Martín-Mora Parra G. Synergy between acceptance of violence and sexist attitudes as a dating violence risk factor. Int J Environ Res Public Health. 2020;17(14):5209. 10.3390/ijerph17145209. PMC7400527 32707658

[20] Linares R, Aranda M, García-Domingo M, Amezcua T, Fuentes V, Moreno-Padilla M. Cyber-dating abuse in young adult couples: relations with sexist attitudes and violence justification, smartphone usage and impulsivity. PLoS One. 2021;16(6):e0253180. 10.1371/journal.pone.0253180. PMC8216513 34153073

[21] União de Mulheres Alternativa e Resposta (UMAR, 2023). ART´THEMIS+. Estudo Nacional sobre a violência no namoro. Novo Estudo 2023 [Cited 2024 2024 April 02]. UMAR. Available from: https://www.cig.gov.pt/wp-content/uploads/2023/02/InfografiaVN_UMAR_2023_Final_pages-to-jpg-0001.png.

[22] Veríssimo AVR, Silva EA, Soares KHD, Amaral ELS, Neto WB, Ludermir AB, et al. Prevalência e fatores associados à violência no namoro entre adolescentes de escola pública. Rev Gaúcha Enferm. 2022;43:e20210170. 10.1590/1983-1447.2022.20210170.pt. 35920525

[23] Farmer C, Shaw N, Rizzo AJ, Orr N, Chollet A, Hagell A, et al. School-based interventions to prevent dating and relationship violence and gender-based violence: systematic review and network meta-analysis. AJPH. 2023;113(3):320–30. 10.2105/AJPH.2022.307153. PMC9932388 36791352

[24] Mineo R, Hofstede A, Newara R, Abdeltawab A-SM, Ndungu F, Rakhawy M. Teen dating violence among Italian high school students: a quantitative study on gender differences. Youth Voice J. 2022;12:3–33. https://iris.unimore.it/retrieve/0a3b5861-dfaf-465c-b2e7-80674d0ae9d5/Mineo%20et%20al.%20YVJ%202022.pdf.

